# AOJNMB enters the Second Decade of Publication

**DOI:** 10.22038/AOJNMB.2022.69339.1483

**Published:** 2023

**Authors:** Seyed Rasoul Zakavi

**Affiliations:** Editor in chief

**Keywords:** AOJNMB, SCIMAGO Journal rank, Editorial

## Abstract

No Abstract is available.

 When AOJNMB was born in May 2013, many were not optimistic about its success. Now, after 10 years of successful on-time publication, it has been indexed in many major indexing banks such as PUBMED central and SCOPUS, and is entering the second decade of its life with a relatively strong position among journals in the field of nuclear medicine. Currently, AOJNMB stands in Q3 of the SCIMAGO journal ranking and outperformed some of the famous national journals in this field (1). 

 SCIMAGO institute uses the SCOPUS database from Elsevier B.V to rank the publications in different categories and provides reliable indices for comparison of journals. SCIMAGO groups journals in four different quartiles and ranks them according to their importance in different disciplines. The first quartile (Q1)

includes the top 25% of journals in a specific discipline and the lowest quartile (Q4) consists of the bottom 25%. For this purpose, it uses a metric named Scimago Journal Rank (SJR), a numeric value that represents the average number of weighted citations received during a selected year per document published in that journal during the previous three years. The Q3 category of the journals in the field of “Radiology, Nuclear Medicine and Imaging” includes many famous names like Annals of Nuclear Medicine, Nuclear Medicine Communications, and Quarterly Journal of Nuclear Medicine (Table 1). In this category, AOJNMB has SJR of 0.325, and stands higher than Nuklearmedizin, Revista Espanola de Medicina Nuclear e Imagen Molecular, and Nuclear Medicine Review (1). 

**Table 1:** SJR of nuclear medicine journals in the category of “Radiology, Nuclear Medicine and imaging” and its quartile group

**Journal Name**		**SJR**	**Journal Name**		**SJR**
**Q1**	Journal of Nuclear Medicine	2.113	**Q3**	Annals of Nuclear Medicine	0.517
	European Journal of Nuclear Medicine and Molecular imaging	2.003		Nuclear Medicine Communications	0.394
	Seminars in Nuclear Medicine	1.092		Asia Oceania Journal of Nuclear Medicine & Biology	0.325
	EJNMMI physics	1.031		Nuklearmedizin	0.269
**Q2**	Journal of Nuclear Cardiology	0.842	**Q4**	Hellenic Journal of Nuclear medicine	0.258
	EJNMMI Research	0.780		Nuclear Medicine Review	0.217
	Clinical Nuclear Medicine	0.520		Medecine Nucleair	0.142

 Another important journal metric that can be used for the comparison of journals and articles is called SNIP. As different fields of research have different potentials to get citations, Source Normalized Impact per Paper (SNIP) is developed to provide a more accurate estimation of impact for any publication. Take a journal in chemistry, for instance, that has the potential to be cited by a wide variety of journals in the field of basic and clinical sciences, and compare it to another journal dedicated to a specific disease like thyroid or a specific technique like Nuclear Medicine that has more limited scope for getting citations. SNIP tries to compensate for the difference in citation potential. The highest SNIP in nuclear medicine goes to the Journal of Nuclear Medicine at 2.41 followed by EJNMMI at 2.39. With a SNIP of 0.561, AOJNMB is thriving to play an important role in the field and is not far away from nuclear medicine communications (SNIP=0.59) for example (2).

 The editors and reviewers of AOJNMB have given higher weight to quality rather than number of the published articles and it can be seen in the current rejection rate of around 40% of submitted manuscripts to AOJNMB (3). This is also reflected in citation metrics of AOJNMB that have been steadily rising since 2014 and we have reached about 240 citations in 2020 (Figure 1). With an h-index of 16 and i10-index of 39, we hope AOJNMB could get a significant impact factor after inclusion in Science Citation Index (4). 

**Figure 1:**
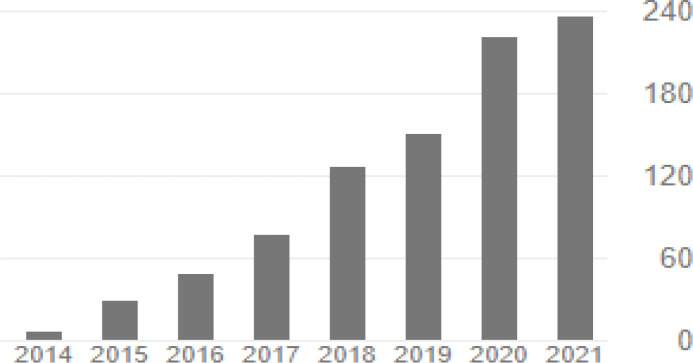
Number of citations of articles published in AOJNMB from 2014 to2021

 The second decade of life of a human being is usually considered the flourishing period. Why not for the AOJNMB? In the first decade of life, AOJNMB has published hundreds of articles in the field of nuclear medicine, physics, and radiopharmacy, and the number of articles per issue has been increasing during the period. With more than 477000 views in total and nearly 1500 downloads per article, AOJNMB has marked excellent visibility. Authors from 28 different countries on five continents have shared their studies with AOJNMB, indicating that we reached out nuclear medicine community throughout the world. The contribution of the countries in AOJNMB publications, however, was not optimal. In the Asia Oceania region, for instance, while Japan, Iran, Australia, and India have published the highest number of articles in the mentioned order, some other countries with great potential like China, South Korea, and Turkey failed to do so (3). As professor Gang Huang took the presidency office of the Asia Oceania Federation of Nuclear Medicine and Biology on July 15, 2021, I look forward to welcoming more manuscripts and reviews from China in the years ahead. Given that AOJNMB is secured a fairly good position among nuclear medicine journals, I am confident it will attract much more interest in the second decade of its publication.

 The authors who shared their study with AOJNMB, and the editors and reviewers who dedicated their precious time and energy to support AOJNMB has been the key to the success of AOJNMB and their efforts cannot be appreciated in words. I should also thank the research deputy of Mashhad University of Medical Sciences that financially supported AOJNMB publication from the very first issue and I hope they could continue their support in the future.

 Finally, at the time of writing this editorial, we are approaching Yalda festival, one of the traditional Persian festivals that celebrate the winter solstice as the triumph of light over darkness. Although the day on December 22nd is just one minute longer than the day before, it is a prelude to the victory of the sun, at least in the northern hemisphere! And it is a unique opportunity for me to sincerely congratulate all authors, reviewers, and readers of AOJNMB, not only for the Yalda festival but also for Christmas and New Year 2023. I sincerely wish all readers of AOJNMB a very prosperous, peaceful, happy, and healthy year ahead. Let us celebrate these festivities and keep our fingers crossed to have a plague-free and violence-free world in the New Year 2023. 
